# Genetic alterations of telomere maintenance pathways in paragangliomas

**DOI:** 10.20945/2359-4292-2026-0050

**Published:** 2026-05-15

**Authors:** Lucas Batini, Eduardo C. Lobato, Felipe Freitas-Castro, Lucas S. Santana, Gustavo F. C. Fagundes, Ana Caroline F. Afonso, Izabel T. Nakamura, Lucas B. Rossetti, Berenice B. Mendonca, Ana Claudia Latronico, Madson Q. Almeida

**Affiliations:** 1 Unidade de Adrenal, Laboratório de Endocrinologia Molecular e Celular LIM25, Divisão de Endocrinologia e Metabologia, Hospital das Clínicas, Faculdade de Medicina da Universidade de São Paulo, São Paulo, SP, Brasil; 2 Laboratório de Hormônios e Genética Molecular LIM42 e Laboratório de Sequenciamento em Larga Escala (SELA), Divisão de Endocrinologia e Metabologia, Hospital das Clínicas, Faculdade de Medicina da Universidade de São Paulo, São Paulo, SP, Brasil; 3 Unidade de Oncologia Endócrina, Instituto do Câncer do Estado de São Paulo (ICESP), Faculdade de Medicina da Universidade de São Paulo, São Paulo, SP, Brasil

**Keywords:** Adrenal paraganglioma, paraganglioma, telomerase, genetics, exome sequencing

## Abstract

**Objective:**

To investigate somatic pathogenic variants (PVs) in the *TERT* promoter region and variants in the *ATRX* gene, both related to telomere maintenance in cancer, in a Brazilian cohort of adrenal and extra-adrenal paragangliomas (PPGLs), and to correlate these with metastatic disease as well as with clinical, radiological, and pathological characteristics.

**Materials and methods:**

The *TERT* promoter was analyzed by automated Sanger sequencing or whole-exome somatic sequencing in a cohort of 79 patients with PPGLs (53 non-metastatic and 26 metastatic), encompassing a total of 81 tumors. *ATRX* was assessed through whole-exome somatic sequencing in 26 patients from this cohort.

**Results:**

Germline PVs in Cluster 1A genes were identified in 28 of 79 patients (35.4%), including 20 patients harboring PVs in the *Succinate Dehydrogenase Complex Iron Sulfur Subunit B gene* (*SDHB*) (25.3%) and 8 patients (10.1%) with PVs in other Cluster 1A genes. Somatic PVs in the *TERT* promoter (c.-124C>T/C228T) were detected in two metastatic PPGLs (2.5% of the total cohort). Three PPGLs (two metastatic), among the 26 patients studied, harbored somatic *ATRX* variants classified as likely benign (11.5%). Somatic PVs in the *TERT* promoter were identified in patients with germline *SDHB* PVs. Among metastatic patients with germline *SDHB* PVs, the frequency of somatic PVs in the *TERT* promoter was 16.7%.

**Conclusion:**

This study expands the understanding of telomere maintenance mechanisms in PPGLs in a Brazilian cohort enriched for *SDHB* alterations. Somatic variants in the *TERT* promoter were associated with aggressive tumor features, such as extra-adrenal location, germline *SDHB* PVs, and metastatic disease.

## INTRODUCTION

Adrenal and extra-adrenal paragangliomas (PPGLs) are neuroendocrine neoplasms derived from chromaffin cells of the adrenal medulla (adrenal PPGLs, formerly named pheochromocytomas) or from sympathetic and parasympathetic ganglia (PPGLs) ^(1,2)^. These tumors have marked clinical heterogeneity and a high association with hereditary syndromes, with germline pathogenic or likely pathogenic variants (PVs) detected in up to 40% of cases ^(3)^. Although most PPGLs are non-metastatic, approximately 15-20% are metastatic, as defined by the presence of metastases in non-chromaffin tissues. Metastatic PPGLs are more prevalent in patients with genetic alterations in the pseudohypoxic cluster, larger tumors (≥ 5 cm), extra-adrenal PPGLs, and those with a noradrenergic or dopaminergic biochemical profile ^(4,5)^.

The classification of PPGLs has recently changed with the 2022 WHO Classification, which designates all PPGLs as paragangliomas: adrenal and extra-adrenal PPGLs referring to the tumors traditionally known as pheochromocytomas and PPGLs, respectively ^(6)^. PPGLs are closely associated with germline PVs, present in approximately 30-40% of cases, and with somatic PVs, identified in up to 30% of tumors ^(7)^. More than 20 susceptibility genes are implicated, broadly categorized into three major molecular clusters: Cluster 1, comprising genes involved in cellular pseudohypoxia signaling, particularly the *Succinate Dehydrogenase Complex Iron Sulfur Subunit B* gene (*SDHB*); Cluster 2, involving genes related to tyrosine kinase-driven signaling pathways; and Cluster 3, involving alterations in the WNT/β-catenin pathway ^(7,8)^. At our institution, genetic testing was performed in 182 patients with PPGLs, the largest Brazilian cohort to date, revealing *SDHB* as the most frequently altered gene (16.5%), a prevalence higher than that reported in other international cohorts ^(7,9,10)^. In addition, a germline *SDHB* exon 1 deletion was responsible for approximately half of these cases, consistent with a Brazilian founder effect, as we have previously demonstrated ^(11)^. In another Brazilian cohort of 115 PPGL patients from a separate institution, 67 had a germline variant identified; the frequency of *SDHB* PVs in this group was roughly 7% (8 patients) ^(12)^.

Pathological mechanisms of telomere elongation can be activated in various cancer types. The two principal mechanisms are *telomerase reverse transcriptase* gene (*TERT*) activation and alternative lengthening of telomeres (ALT) ^(13)^. Most human cancers display some degree of *TERT* hyperactivity, which is the primary mechanism underlying replicative immortality ^(13,14)^. The predominant genetic mechanism for pathological *TERT* activation involves somatic PVs at hotspot sites in the *TERT* promoter (C228T and C250T), which lead to increased gene transcription and are associated with metastatic behavior in PPGLs ^(15)^. Additionally, the ALT mechanism has been strongly linked to somatic variants in the *ATRX chromatin remodeler* gene (*ATRX*). A previous study involving PPGL 200 patients demonstrated that the ALT phenotype correlates with *ATRX* variants and metastatic disease, highlighting *ATRX* alterations as significant prognostic markers in this tumor type ^(15)^. Interestingly, somatic PVs in the *TERT* promoter and *ATRX* are more frequently observed in *SDHB-*related PPGLs ^(15,16)^.

In a multi-omics study, *ATRX* and *TERT* alterations were also associated with metastatic PPGLs, together with high tumor mutational burden, high microsatellite instability, and Cluster 1A PVs ^(17)^. Nevertheless, genetic alterations in telomere maintenance pathways have not yet been investigated in a Brazilian PPGL cohort. Given the distinct genetic landscape of PPGLs among different ethnicities, including the high prevalence of *SDHB* PVs in our Brazilian cohort, we aimed to investigate somatic PVs in the *TERT* promoter and *ATRX* in PPGLs from Brazilian patients.

## MATERIALS AND METHODS

### Ethical aspects and inclusion criteria

This study was conducted in accordance with the ethical principles outlined in the Declaration of Helsinki ^(18)^. The project was approved by the Research Ethics Committee of Hospital das Clínicas da Faculdade de Medicina da Universidade de São Paulo (approval no. 5.156.162) and the Instituto do Câncer de São Paulo Octavio Frias de Oliveira (approval no. 1448/19). Written informed consent was obtained from all participants or their legal guardians.

In this study, 81 tumors from 79 patients with PPGLs were evaluated, including one case of a metastasis paired with its primary tumor and one patient with two primary tumors. Among these, 53 patients had non-metastatic PPGL, and 26 had metastatic PPGL. The biochemical and imaging diagnosis of PPGLs followed the recommendations of the Endocrine Society ^(1)^. Metastatic disease was defined as the presence of a neuroendocrine tumor in non-chromaffin tissue, confirmed by biopsy or functional imaging ^(131^I-MIBG, PET-CT with ^68^Ga-DOTATE, or octreoscan) ^(6,19)^. Staging was performed using the TNM system for PPGLs developed by the American Joint Committee on Cancer ^(19)^. The main inclusion criterion was the availability of blood and tumor DNA for prior genetic investigation. All PPGL cases included had a confirmed histopathological diagnosis. Cases without a genetic diagnosis were defined as those lacking PVs in genes previously associated with PPGL pathogenesis, according to the criteria of the American College of Medical Genetics and Genomics (ACMG) and the Association for Molecular Pathology (AMP) ^(20)^.

### Study cohort and sample collection

The cohort of 79 PPGL patients had previously undergone Sanger sequencing, targeted gene panel sequencing, and/or whole-exome sequencing (WES) ^(10)^. Germline DNA was extracted from peripheral blood using the salting-out method. Tumor tissue samples obtained during routine surgery were stored in liquid nitrogen prior to nucleic acid extraction, totaling 68 cases. In 13 cases where only formalin-fixed, paraffin-embedded (FFPE) tumor tissue blocks were available, blocks containing more than 90% tumor content were selected by an experienced adrenal tumor pathologist based on hematoxylin and eosin-stained slides. Two 20 µm sections were obtained from each selected FFPE block for DNA extraction.

### DNA extraction and quality assessments

DNA was extracted from fresh tissue samples using the AllPrep DNA/RNA/miRNA Mini Kit (Qiagen, Germany). DNA from FFPE tissues was extracted using the QIAamp DNA FFPE Advanced kit (Qiagen, Hilden, Germany). For FFPE samples, a protocol optimized for next-generation sequencing was employed, treating DNA with uracil-N-glycosylase to minimize artifactual nucleotide changes due to cytosine deamination. The quality and concentration of extracted DNA were assessed using a NanoDrop spectrophotometer.

### PCR Amplification and Sanger sequencing of the *TERT* promoter

The *TERT* promoter region (chr5: 1295151-1295313) was amplified with previously described oligonucleotides ^(15)^ (Primer F: GTCCTGCCCCTTCACCTT; Primer R: CAGCGCTGCCTGAAACTC), generating a 163 bp amplified fragment for sequencing. PCR reactions were performed using 10-15 pmol/µL of each oligonucleotide and 200 µM deoxynucleotide triphosphate in a final volume of 25 µL. The PCR protocol consisted of 35 cycles with an annealing temperature of 59 °C ^(15)^.

*TERT* promoter was performed with the KAPA HiFi Hot Start ReadyMix kit (Kapa Biosystems). The concentration of cDNA or DNA in the PCR products was estimated by comparison to molecular weight markers of known concentrations (ΦX) on agarose gel. The resulting amplicons underwent enzymatic purification using ExoSap-it (Life Technologies, Carlsbad, CA, USA), incubated at 37 °C for 15 min, followed by enzyme inactivation at 80 °C for 15 min.

Sequencing reactions were performed using the ABI PrismTMBigDyeTerminator kit (Applied Biosystems, USA), with 10-100 ng of PCR product, adjusted according to fragment size. Sequencing was performed on an ABI Prism Genetic Analyzer 3130xl (Applied Biosystems, USA). Electropherograms were read using the Sequencher v. 4.10.1 software (GeneCodes Corporation, USA), always in comparison with the reference gene sequence and an electropherogram from a normal individual.

### Whole-exome sequencing and bioinformatics analysis

In addition to Sanger sequencing, analysis of the *TERT* promoter region and *ATRX* somatic variants was performed in 26 samples previously investigated by WES ^(10)^. The WES was conducted using the DNA nanoball sequencing platform (DNBSEQ, BGI Genomics Europe, Denmark) after somatic exome enrichment with KAPA HyperExome Probes (USA), ensuring high-quality sequencing characterized by a low duplicate rate and deep coverage. Raw FASTQ files were demultiplexed and processed through a pre-mapping pipeline to assess integrity and quality and to clean sequencing artifacts and barcodes. Grommer1 was used to verify FASTQ file integrity (ASCII encoding offset, Phred score calculation, read pair identification, etc.) while FastQC assessed sequencing quality (number of reads, read length distribution, read quality, etc.). Sequence filtering and trimming were performed using BBDuk (BBTools package). Processed FASTQ files were submitted to a reference-based (GRCh37/hg19) mapping approach using BWA-MEM alignment algorithm, and duplicate reads were marked or removed using Picard2. Mapping quality and coverage of target regions were assessed with Qualimap3 (Qiagen, Germany) ^(10)^. Across the 26 DNA samples, ≥89.56% (mean: 95.17%) of the reference achieved a minimum depth of 20× coverage.

### Variant identification and validation

The *TERT* promoter region (chr5: 1295151-1295313) was manually inspected for hotspot variants using the Integrative Genomics Viewer software ^(21)^. The two main *TERT* hotspots, chr5: 1295228 and chr5: 1295250, had mean coverage depths of 111.17× and 96.88×, respectively. *TERT* promoter variants selected for further analysis had coverage >20×, allelic balance >10%, no significant strand bias, and adequate base and mapping quality. Visualization filters were applied to ensure that detected variants were not artificial due to strand bias or poor read quality. All WES-identified *TERT* promoter variants were confirmed by Sanger sequencing. *ATRX* single nucleotide variants and copy number variants were analyzed using the Franklin platform (Qiagen, Germany). Variants were included if they showed at least 20× coverage, allelic balance >10%, and were located within the available WES coverage region.

### Statistical analysis

Descriptive statistics were reported as absolute and relative frequencies for qualitative variables and as means with standard deviations or medians with interquartile ranges, for quantitative variables. Quantitative variables were compared between two groups using the t-test if the assumption of normality was met, otherwise, the Mann-Whitney U test was applied. Normality was assessed with the Shapiro-Wilk test and equality of variances with the Brown-Forsythe test. Categorical variables were compared using Pearson’s chi-square test or Fisher’s exact test, as appropriate. Statistical analyses were performed using the JASP software v. 19.0 (University of Amsterdam, Netherlands). All hypothesis tests were two-tailed, with a significance level set at 0.05.

## RESULTS

### Cohort characteristics

In the cohort of 79 patients, the median age at diagnosis was 33 years (range: 10-72 years). The cohort comprised 50 females (63.3%). Adrenal PPGL was diagnosed in 45 patients, whereas 28 patients developed extra-adrenal PPGL. Multiple PPGLs were observed in 23 patients (29.1%). Metastases were diagnosed in 26 patients (32.9%). TNM staging distribution was: 18 patients at stage I (22.7%), 27 at stage II (34.2%), 12 at stage III (15.2%), and 15 at stage IV (19.0%). TNM staging could not be assessed in seven patients. The mean maximum tumor diameter by imaging measured 5.9 cm for adrenal PPGLs and 6.2 cm for extra-adrenal PPGLs (**Table 1**).

**Table 1:** Clinical, biochemical, and evolutive characteristics of patients with adrenal paragangliomas and paragangliomas who underwent genetic testing

Patients		n = 79
Age at diagnosis (years)^a^		33 (10-72)
Female sex, n (%)		50 (63.3)
Adrenal paragangliomas, n (%)		45 (57.0)
	Bilateral	13 (28.9)
Paragangliomas, n (%)		28 (35.4)
	Abdominal	22 (64.7)
	Thoracic	1 (3.0)
	Genitourinary	1 (3.0)
	Head and neck	6 (17.6)
Multiple paragangliomas^b^		23 (29.1)
Hormone profile, n (%)		
	Non-functioning	10 (12.7)
	Adrenergic	3 (3.8)
	Noradrenergic	28 (35.4)
	Mixed	16 (20.2)
	Unknown	15 (17.8)
	Unavailable	8 (10.1)
Largest diameter at imaging (cm)^c^		
	Adrenal paraganglioma	5.9 ± 3.1
	Paraganglioma	6.2 ± 3.0
Metastatic patient, n (%)		26 (32.9)
TNM staging, n (%)		
	1	18 (22.7)
	2	27 (34.2)
	3	12 (15.2)
	4	15 (19.0)
	Unavailable	7 (8.9)

a Median (range).

b Bilateral adrenal paraganglioma or adrenal paraganglioma with paraganglioma or multiple paragangliomas.

c Mean ± standard error.

### Genetic findings

**Figure 1:**
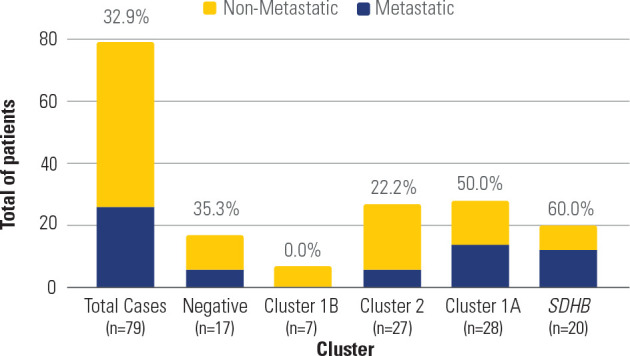
Total of patients and proportion of metastatic tumors by genetic cluster. Note the higher proportion of metastases in patients in Cluster 1A, especially those with *SDHB* pathogenic variants.

Germline PVs in Cluster 1A genes were identified in 28 patients (35.4%), including 20 harboring *SDHB* PVs (25.3%) and 8 (10.1%) harboring PVs in other Cluster 1A genes (including additional *SDHx* genes, *FH, SLC25A11*, and *DSLT*). PVs in Cluster 1B genes (*VHL* and *EPAS1*) were identified in 7 patients (8.9%), and PVs in Cluster 2 genes (e.g., *NF1, MAX, RET, HRAS*, and *TMEM127*) were identified in 27 patients (34.2%). Seventeen patients (21.5%) had negative genetic testing. The distributions, as well as the percentage of metastatic cases within each cluster, are presented in **Figure 1.**

**Figure 2:**
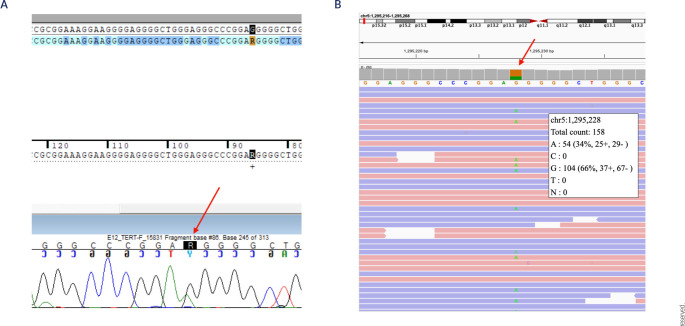
Identification of the somatic *TERT* promoter variant. (**A**) Sanger sequencing chromatogram of the *TERT* promoter region in a somatic PPGL sample displaying the c.-124C>T variant detected in DNA as G>A (arrow). (**B**) Integrative Genomics Viewer visualization of aligned Whole Exome Sequencing data from the same tumor. The variant (chr5:1,295,228 G>A) is present at a 34% variant allele frequency with a total read depth of 158× (arrow). Reads are color-coded by strand to demonstrate a balanced distribution, supporting the technical reliability of the call and the absence of strand bias.

The somatic *TERT* promoter PV C228T was detected in two PPGLs, representing 2.5% of total cases in the cohort. Both patients harbored germline *SDHB* PVs and had metastatic behavior. *TERT* promoter PVs were detected by automated Sanger sequencing and somatic exome analysis (**Figure 2**). The somatic *TERT* promoter PV C250T was not identified in any patient.

The *ATRX* gene was evaluated by WES in 26 PPGLs, 21 of which belonged to Cluster 1A. *ATRX* variants were identified in three tumors (11.5%), all classified as likely benign (LB). The corresponding cases are briefly reported below, although these variants are not likely related to pathogenic alterations in telomere maintenance pathways. Notably, in a case in which both the primary tumor and the corresponding metastatic lesion were simultaneously analyzed, no variants were identified in either the *TERT* promoter or *ATRX*.

### Clinical, biochemical, and radiological characteristics

Clinical, biochemical, and radiological characteristics of tumors harboring genetic alterations in the *TERT* promoter or *ATRX* are described below and summarized in **Table 2**. The first patient whose tumor harbored a C228T PV in *TERT* (*TERT:c.-124C>T*) was a male with a germline *SDHB* PV (*SDHB:*c.591delC; p.Ser198Alafs*22), a frameshift variant resulting in a truncated protein. At age 18, he presented with resistant hypertension despite clinical management. Although he exhibited symptoms and an abdominal mass identified by ultrasound at that time, definitive diagnosis and treatment were only established at age 33. He was diagnosed with an abdominal PPGL exhibiting a noradrenergic secretion profile, consistent with the clinical symptoms he manifested. Surgical resection revealed a 13.5 cm PPGL, staged as T3NxM0 (stage III), with a Pheochromocytoma of the Adrenal Gland Scaled Score (PASS) of 2. The patient remains under follow-up and treatment.

**Table 2:** Clinical, histopathological, and genetic characteristics of tumors with somatic findings in *TERT* promoter or *ATRX*

Germline driver	Somatic variant	ACMG	Hormone profile	Age at diagnosis (years)	Local	Size (cm)^a^	Metastasis
*SDHB* (C.591delC / p.Ser198Alafs^*^22)	*TERT:c.-124C>T*	P	NE	33	PGL Abd	13.5	Yes
*SDHB* (c.293G>A/ p.Cys98Tyr)	*TERT:c.-124C>T*	P	NE	57	PGL GU	8	Yes
*SDHB* (c.exon 1 del / p.?)	*ATRX:* c.20+22T>C	LB	NF	25	PGL HN	Unknown	Yes
*SDHB* (c.688C>T / p.Arg230Cys)	*ATRX:* c.1633C>G	LB	NE	29	PGL Abd	14.7	Yes
Unknown	*ATRX:* c.-88C>T	LB	NE	34	Ad	9.5	No

SDHB: succinate dehydrogenase complex iron sulfur subunit B; FH: fumarate hydratase gene; P: pathogenic; VUS: variant of uncertain significance; LB: likely benign; NE: norepinephrine; NF: non-functioning; Abd: abdominal; GU: genitourinary; HN: head/neck; Ad: adrenal paraganglioma; PGL, paraganglioma; ACMG: American College of Medical Genetics and Genomics Classification.

a The largest diameter at imaging.

The second patient, who harbored the same *TERT* PV, was a male with a germline *SDHB* PV (*SDHB:*c.293G>A; p.Cys98Tyr), a missense variant with strong familial segregation, as several relatives with the same PV had also been diagnosed with PPGLs. His tumor was an 8 cm bladder PPGL, characterized by paroxysmal hypertension triggered by bladder distension, and a noradrenergic secretion profile consistent with his clinical history and symptoms. He had been followed by the cardiology service for nearly 12 years due to heart failure with reduced ejection fraction before being diagnosed with PPGL at age 57. At diagnosis and treatment, the tumor was staged as T2NxM1a (stage IV) with bone metastases and a PASS score of 5. Two months later, he was hospitalized for an ischemic stroke and died shortly thereafter. Notably, both the *TERT* somatic PV and *SDHB* germline PV co-occurred with a heterozygous germline *TP53* PV (*TP53:*c.1010G>A; p.Arg337His), which may have contributed to the aggressive phenotype and poor prognosis.

Somatic *ATRX* variants were identified in three tumors, all classified as LB. The first patient, diagnosed at age 25 with a nonfunctioning cervical PPGL, subsequently developed bone and lung metastases and was staged as T2NxM1c (stage IV). Genetic analysis revealed a germline *SDHB* PV (exon 1 deletion; seq[GRCh37/hg19] 1p36.13(17375250_17390928)x1) and a somatic LB *ATRX* variant (*ATRX:*c.20+22T>C).

The second patient was a male who presented with a 14.7 cm abdominal PPGL with a noradrenergic secretion profile and distant metastases to bone and lymph nodes. He was staged as T2N1M1a (stage IV) and remains under follow-up with the endocrinology service. Genetic testing identified a germline *SDHB* PV (*SDHB:*c.688C>T; p.Arg230Cys) and a somatic LB *ATRX* variant (*ATRX:*c.1633C>G).

The third patient had a 9.5 cm left adrenal PPGL with a noradrenergic secretion profile, diagnosed and treated at age 34. The tumor was staged as T2NxM0 (stage II), with a PASS score of 5. No germline PVs were previously detected, and the somatic *ATRX* variant was classified as LB (*ATRX:*c.-88C>T).

### Correlation of genetic data with clinical and histopathological findings

A significant association was observed between the presence of PVs in Cluster 1A genes and metastases, with a higher rate of metastasis in this cluster than others (50% vs. 23.5%, respectively; *p* = 0.017) (**Figure 1**). Similarly, tumors harboring germline *SDHB* PVs showed a higher prevalence of metastatic disease than tumors without *SDHB* PVs (60% vs. 23.7%, respectively; *p* = 0.003). Among metastatic cases, somatic *TERT* promoter alterations were detected in 2 out of 26 cases (7.7%). When only considering metastatic tumors harboring germline *SDHB* PVs, *TERT* promoter alterations were observed in 2 of 12 cases (16.7%). *ATRX* variants were detected in 2 of the 13 metastatic tumors subjected to WES (15.4%), two LB variants. When restricting the analysis to metastatic tumors with germline *SDHB* PVs, *ATRX* variants were identified in 2 of 12 cases (16.7%).

## DISCUSSION

This study presents the first systematic evaluation of genetic alterations in telomere maintenance pathways in a Brazilian cohort of PPGLs, characterized by a high prevalence of *SDHB* PVs. Consistent with previous reports, we confirmed a strong association between Cluster 1A alterations, particularly germline *SDHB* PVs, and aggressive disease features, including metastatic presentation ^(7,17)^. The high frequency of *SDHB* alterations observed in our cohort, largely driven by a recurrent exon 1 deletion, reinforces the relevance of population-specific genetic backgrounds and founder effects in shaping the molecular epidemiology of PPGLs ^(9-11,22)^.

In PPGLs, PVs in the *TERT* promoter and in *ATRX* represent key markers of pathological telomere-elongation mechanisms that promote replicative immortality ^(14)^. Telomeres are repetitive nucleoprotein structures at chromosome ends whose integrity is essential for chromosomal stability and proper regulation of cell division. Progressive telomere shortening, resulting from the incomplete replication of linear DNA molecules by DNA polymerase, is evolutionarily counteracted by telomerase, a ribonucleoprotein complex whose catalytic core adds telomeric repeats to chromosome ends ^(23)^.

Our findings further suggest that somatic alterations in the *TERT* promoter, although infrequent, occur preferentially in *SDHB*-related PPGLs and are associated with metastatic behavior. Both tumors harboring *TERT* promoter C228T variants in our cohort were PPGLs, displayed a noradrenergic biochemical phenotype, and developed metastatic disease. These observations are consistent with prior studies linking *TERT* promoter PVs to telomerase activation, replicative immortality, and poor prognosis in PPGLs and other solid tumors ^(15)^. Notably, the absence of the C250T variant in our cohort reflects its lower prevalence reported in PPGLs compared with other malignancies ^(24,25)^.

In addition to *TERT* alterations, *ATRX* variants were identified in a subset of tumors, predominantly within Cluster 1A. Most patients harboring *TERT* or *ATRX* variants had tumors larger than the cohort’s mean diameter (5.9 cm for adrenal PPGLs and 6.2 cm for extra-adrenal PPGLs) and had a predominantly noradrenergic profile. These two characteristics are associated with metastatic disease, as described herein. Furthermore, these patients had worse outcomes than others in the cohort, with most developing metastatic disease (4 out of 5), further supporting the possible relationship between these variants and prognostic markers.

Although *ATRX* variants were classified as LB according to current ACMG criteria, their presence was associated with larger tumor size and a higher prevalence of metastatic disease. This observation supports previous evidence linking *ATRX* dysfunction to the ALT phenotype and tumor aggressiveness in PPGLs ^(15-17,26)^. Importantly, variant classification frameworks are largely optimized for germline variants, and their applicability to somatic alterations, particularly in genes involved in chromatin remodeling and telomere biology, remains limited. Thus, the biological impact of somatic *ATRX* variants may be underestimated when relying solely on conventional pathogenicity categories. Nevertheless, we acknowledge that further investigation (including functional studies and additional gene expression assays) is necessary to validate these specific *ATRX* variants and their possible association with the ALT phenotype.

The association between telomere-related genetic alterations and adverse clinical features was further supported by our imaging and clinical analyses. Paragangliomas harboring *TERT* promoter or *ATRX* variants exhibited larger diameters on imaging and a higher prevalence of metastatic disease compared with tumors lacking these alterations. These findings suggest that telomere maintenance dysregulation, especially through *TERT* promoter PVs, may contribute not only to metastatic potential but also to enhanced tumor growth. Although germline *SDHB* PVs represent the strongest genetic predictor of metastatic disease, approximately 50% of patients with *SDHB*-mutated PPGLs do not develop metastases ^(27-29)^. From a clinical perspective, identifying additional molecular alterations may help refine risk stratification, especially in patients with *SDHB*-related disease, for whom predicting malignant behavior remains challenging.

A study evaluating 79 individuals with germline *SDHB* PVs, including 34 with metastatic disease, found *TERT* promoter PVs in 14 patients (41.2% of metastatic cases) ^(16)^. These findings were not replicated in a larger French cohort of 200 PPGLs, which reported a 3.5% prevalence for somatic *TERT* promoter PVs ^(15)^. Among 11 patients with both germline *SDHB* PVs and metastatic disease, 5 (41.7%) harbored *TERT* promoter variants, compared with 18% in our cohort ^(15)^. Although limited by sample size, this discrepancy warrants further investigation in larger and independent cohorts.

This study has several strengths. It represents the first dedicated analysis of telomere maintenance-related genetic alterations in a Brazilian PPGL cohort, a population with a distinct genetic background and a high prevalence of *SDHB* PVs. Comprehensive molecular characterization, combining germline testing, somatic *TERT* promoter sequencing, and WES for *ATRX*, enabled an integrated evaluation of telomere-related pathways in relation to clinical, biochemical, and radiological features. In addition, the use of standardized diagnostic criteria, centralized pathological review, and detailed phenotypic annotation strengthened the robustness of genotype-phenotype correlations. Nevertheless, some limitations must be acknowledged. The retrospective design and the relatively small number of tumors harboring *TERT* promoter or *ATRX* alterations limited statistical power and precluded multivariable analyses. Furthermore, functional validation of *ATRX* variants and direct assessment of telomere length or ALT phenotype were not performed, preventing definitive conclusions regarding causality. Lastly, although our cohort is enriched for *SDHB*-related disease, this may limit the generalizability of the findings to populations with different genetic architectures.

In conclusion, our study expands the understanding of telomere maintenance mechanisms in PPGLs within a Brazilian population enriched for *SDHB* alterations. Somatic variants in the *TERT* promoter were associated with aggressive tumor features, such as extra-adrenal location, germline *SDHB* PVs, and metastatic disease. These findings underscore the importance of integrating telomere-related genetic alterations into the molecular characterization of PPGLs and support their potential role as prognostic markers.

## Data Availability

datasets related to this article will be available upon request to the corresponding author.
